# Transcriptome analysis of northern elephant seal (*Mirounga angustirostris*) muscle tissue provides a novel molecular resource and physiological insights

**DOI:** 10.1186/s12864-015-1253-6

**Published:** 2015-02-08

**Authors:** Jane I Khudyakov, Likit Preeyanon, Cory D Champagne, Rudy M Ortiz, Daniel E Crocker

**Affiliations:** Department of Biology, Sonoma State University, 1801 E Cotati Ave, Rohnert Park, CA 94928 USA; Michigan State University, Microbiology and Molecular Genetics, 567 Wilson Rd, East Lansing, MI 48824 USA; National Marine Mammal Foundation, Conservation and Biological Research Program, 224 0Shelter Island Drive, San Diego, CA 92106 USA; University of California, Merced, School of Natural Sciences, 5200 North Lake Rd, Merced, CA 95343 USA

**Keywords:** Transcriptome, *de novo* assembly, Pinniped, Stress, Cloud computing

## Abstract

**Background:**

The northern elephant seal, *Mirounga angustirostris*, is a valuable animal model of fasting adaptation and hypoxic stress tolerance. However, no reference sequence is currently available for this and many other marine mammal study systems, hindering molecular understanding of marine adaptations and unique physiology.

**Results:**

We sequenced a transcriptome of *M. angustirostris* derived from muscle sampled during an acute stress challenge experiment to identify species-specific markers of stress axis activation and recovery. *De novo* assembly generated 164,966 contigs and a total of 522,699 transcripts, of which 68.70% were annotated using mouse, human, and domestic dog reference protein sequences. To reduce transcript redundancy, we removed highly similar isoforms in large gene families and produced a filtered assembly containing 336,657 transcripts. We found that a large number of annotated genes are associated with metabolic signaling, immune and stress responses, and muscle function. Preliminary differential expression analysis suggests a limited transcriptional response to acute stress involving alterations in metabolic and immune pathways and muscle tissue maintenance, potentially driven by early response transcription factors such as *Cebpd*.

**Conclusions:**

We present the first reference sequence for *Mirounga angustirostris* produced by RNA sequencing of muscle tissue and cloud-based *de novo* transcriptome assembly. We annotated 395,102 transcripts, some of which may be novel isoforms, and have identified thousands of genes involved in key physiological processes. This resource provides elephant seal-specific gene sequences, complementing existing metabolite and protein expression studies and enabling future work on molecular pathways regulating adaptations such as fasting, hypoxia, and environmental stress responses in marine mammals.

**Electronic supplementary material:**

The online version of this article (doi:10.1186/s12864-015-1253-6) contains supplementary material, which is available to authorized users.

## Background

Transcriptomics can vastly improve our understanding of organismal physiology, ecology, and evolution on a large-scale molecular level in both model and non-model systems [1,2]. By comparing abundance of all mRNA transcripts present in tissues between distinct physiological states, transcriptomics has the potential to elucidate the myriad genes and pathways driving processes such as development, fasting, and hibernation [3-5], or responses to environmental change, disease, and other perturbations [6,7]. The fields of stress and conservation physiology especially have much to gain from non-targeted transcriptomics tools, as the molecular bases of organismal responses to altered environmental states and human activity are still not well-understood, especially in wild animals [8,9].

Advancements in sequencing technologies and computational tools are now facilitating sophisticated genomics and transcriptomics studies in non-model organisms [10]. While the cost of sequencing is becoming less prohibitive, data analysis remains a challenge for many biologists, mainly due to limited computational resources [11]. Robust *de novo* assemblers, data reduction tools, and cloud computing are beginning to make sequencing data analysis more approachable for bench and field scientists [12-14]. Despite these improvements, sequence-based resources are still lacking for many non-model species such as marine mammals, hampering molecular understanding of unique adaptations and physiology. Only a handful of marine mammal genomes have been sequenced, annotation remains a challenge, and few transcriptomes are available [15-22].

The northern elephant seal (*Mirounga angustirostris*) is one of the most extensively studied pinniped species that has contributed greatly to our understanding of fasting and diving physiology. Ease of accessibility and amenability to research manipulations make this animal one of the few marine mammal study systems in which functional physiological experiments are feasible [23,24]. Elephant seal life history is characterized by prolonged terrestrial fasts during which animals incur extremely high energy expenditures necessary for breeding and molting [25]. Fasting metabolism relies almost entirely on lipolysis and is characterized by insulin resistance and hyperglycemia, making this species a non-traditional model of metabolic syndrome [23,26]. The elephant seal is also a model of oxidative stress tolerance as animals are routinely exposed to hypoxia during deep dives and apnea but do not incur tissue damage due to high antioxidant capacity [27-29]. Elevated carbon monoxide levels in the blood may also protect elephant seals from oxidative stress, although the mechanisms for this are currently unknown [30]. Elucidating the molecular bases of these adaptations can greatly increase our understanding of marine mammal physiology as well as human pathologies related to metabolism and oxidative stress. However, molecular studies in elephant seals have been limited to small subsets of highly conserved genes in the absence of unique genomic information [31-34]. Transcriptomics can rapidly generate *M. angustirostris*-specific reference sequence in the absence of a genome, enabling large-scale gene discovery in physiological and ecological contexts. This approach also facilitates phylogenomic analyses and can improve genome annotation for other marine mammal species [1,35,36].

We present a reference transcriptome for *M. angustirostris* muscle tissue collected from juvenile animals undergoing a stress challenge experiment. Stress hormones (i.e. glucocorticoids such as cortisol) released by the hypothalamic-pituitary-adrenal (HPA) axis serve an adaptive role in elephant seal physiology by maintaining fasting metabolism and promoting life history transitions [37-40]. However, elevated HPA axis activity in response to environmental disturbance may become pathological, resulting in reduced fecundity and survival, a key conservation issue for species of concern [41]. We are interested in understanding the physiological differences between adaptive and maladaptive stress responses. Downstream effectors of HPA axis activity are relatively unknown in derived mammals such as phocid seals, hindering development of species-specific molecular tools for studying stress physiology. To address this resource gap, we examined global transcriptional changes in elephant seal muscle, a metabolically active target tissue, in response to an acute stress challenge. We stimulated the HPA axis by administering slow-release adrenocorticotropic hormone (ACTH) to juvenile seals, which activates endogenous cortisol release and allows sustained stimulation of the hormone axis [42]. Manipulation and sampling were conducted under dissociative anesthesia to avoid confounds of psychological stress. Prior studies have shown this immobilization procedure does not result in activation of the HPA axis [24]. Tissue samples were collected immediately prior to ACTH administration and 2 and 24 hours post-procedure, representing baseline, acute stress, and stress recovery states. The transcriptome assembly includes samples from all three conditions to capture transcripts expressed during both native and stressed physiological states.

We employed a user-friendly data analysis pipeline (khmer-protocols, see Methods) to perform *de novo* transcriptome assembly and annotation entirely in the cloud [43]. We assembled 1.6 gigabases into 522,699 transcripts, of which 68.70% were annotated using mouse, human, and dog reference sequences. This generated 25,674 annotated transcript families that represent a novel resource for physiological studies in this marine mammal study system. Genes in the reference transcriptome that mapped to functional pathways are involved in insulin signaling and lipid metabolism, molecular pathways in cancer, muscle tissue maintenance, and immune response to pathogens. Preliminary expression analysis found that transcripts altered during an acute stress response in elephant seals, such as CCAAT/enhancer binding protein-δ, are mainly involved in metabolic and immune function. Further investigation of the specific transcriptional response to experimental stress manipulation will yield significant insights into molecular underpinnings of organismal stress response and recovery.

## Results and discussion

### Transcriptome sequencing and assembly

Muscle tissue for transcriptome sequencing was collected from three juvenile northern elephant seals at three time points during an acute stress challenge experiment: before ACTH injection (“0 hr”), 2 hours after injection (“2 hr”), and 24 hours (“24 hr”) after injection. Libraries from the nine samples were pooled and paired-end sequenced by Illumina HiSeq 2500, generating 256 million reads, 25.6 billion total bases, and 66.3 GB of data. Raw sequencing data were deposited at NCBI Sequence Read Archive under study accession [SRP045540]. All data analysis was conducted in the cloud using Amazon Elastic Compute Cloud service x-large and 2x-large machines [43]. The 2x-large machine, which offers 34 GB of memory, was only used for assembly, while the x-large (15 GB) was sufficient for all other analysis steps. We used the Eel Pond mRNAseq Protocol, a user-friendly standalone pipeline for complete transcriptome analysis from quality trimming and data normalization, through assembly, annotation, and expression analysis (https://khmer-protocols.readthedocs.org/).

Raw sequence files were trimmed to remove adapter sequence contamination and low-quality sequences (Trimmomatic [44], Fastx toolkit). Adapter and quality filtering reduced the amount of data from 66.3 GB to 23.0 GB and improved sequence quality scores as determined by FastQC. We found that up to 20% of each paired-end sequence file contained an overrepresented sequence homologous to myoglobin of Weddell seal (score: 79.8, e-value: 2e-12), grey seal (score: 83.8, e-value: 1e-13), and domestic dog (score: 63.9, e-value: 1e-07). Muscle myoglobin protein content is known to be elevated in phocid seals compared to terrestrial species [45], and here we demonstrate that myoglobin homologs are highly overrepresented in the elephant seal transcriptome.

High-throughput RNA sequencing produces a vast amount of data in order to provide sufficient coverage of low-abundance transcripts. This results in significant redundancy of the most abundant transcripts, compounding sequencing errors, complicating assembly, and increasing computing time. Digital normalization (diginorm) removes highly redundant sequences while retaining read complexity and low abundance transcripts [13]. This decreases computational memory needed for assembly without losing valuable biological information. A single round of diginorm was applied to trimmed sequencing files, removing 92.28% of sequence and reducing the total amount of data to 5.2 GB (19.80 million sequences), which enabled cloud-based assembly in 31 hours.

Transcriptome assembly was conducted using Trinity, a de Bruijn graph-based *de novo* assembler that has shown high performance in recovering full-length transcripts and splice isoforms [12,46,47]. Trinity assembled 1.6 gigabases into 522,699 transcripts and 164,966 Trinity components (“gene families”) with 50.88 percent GC content. The mean, median and N50 contig lengths were 3117 bp, 2298 bp, and 5501 bp, respectively (Table 1). The raw assembly is available at http://athyra.ged.msu.edu/~preeyano/seal/Mirounga_raw.fa.gz. While the basic assembly statistics provided by Trinity are commonly used to estimate assembly quality, they are derived from genome-based metrics that do not take into account uneven sequencing coverage and presence of isoforms [47].

**Table 1:** ***Mirounga angustirostris*** **transcriptome assembly statistics**

Total sequenced bases	25.6 billion
Total assembled bases	1.6 billion
Number of transcripts	522,699
Number of components	164,966
Mean contig length (bp)	3,117
Median contig length (bp)	2,298
Contig N50 (bp)	5,501

An alternative quality metric is the percentage of raw reads mapping back to the assembly. This provides an estimation of assembly completeness, an important consideration for downstream analyses using the assembly as a scaffold for read mapping and expression analysis [48]. We used bowtie [49] to align quality-trimmed reads from all nine samples to the assembly. We found that in all sample reads, 92.62% of left and 92.55% of right reads could be mapped back to the assembled transcriptome, with 86.60% proper pairs mapped for a representative sample. Unmapped sequences may represent poor quality reads, incomplete transcripts, or read orphans.

Transcriptome assemblies vary widely by sequencing platform, read length, coverage, and assembly method, so there is still little consensus on what constitutes a “high-quality” *de novo* assembly. It has been suggested that annotation-based metrics are most informative as they estimate the number of real genes that can be recovered from the assembly [50].

### Transcriptome annotation

We annotated the elephant seal transcriptome by searching for best BLAST hits (homologs) and reciprocal best hits (orthologs) between elephant seal and mouse (*Mus musculus*), human (*Homo sapiens*), and dog (*Canis lupus familiaris*) protein sequences. Mouse sequence was used for continuity in the Eel Pond mRNAseq protocol and to obtain Entrez gene IDs for pathway analysis. Human protein sequence was used for annotation due to its completeness. Dog protein sequence was used to identify carnivore-specific genes that are not represented in mouse or human genomes [48,51].

**Figure 1:**
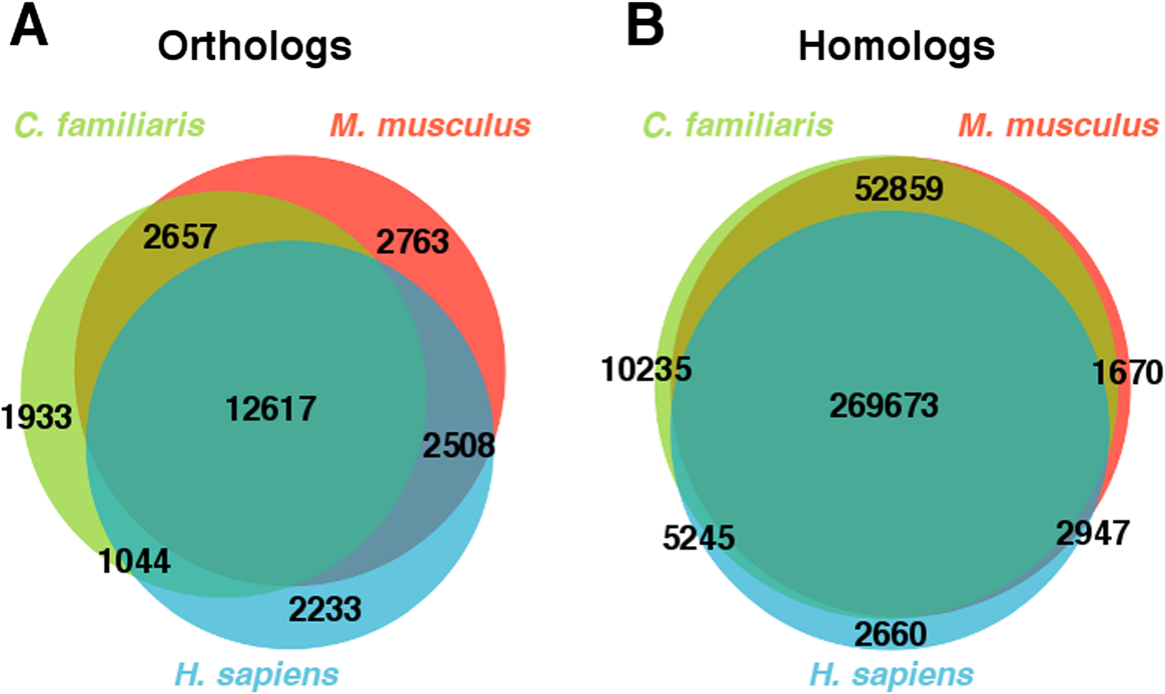
***M. angustirostris*** **(A) orthologs and (B) homologs of mouse (** ***M. musculus*** **, red), human (** ***H. sapiens*** **, blue), and domestic dog (** ***C. familiaris*** **, green) genes.**

Annotation using mouse sequence produced a total of 347,694 (66.52% of total assembled) annotated transcripts in 20,171 annotated transcript families. We found 20,545 and 327,149 *M. angustirostris* orthologs and homologs of mouse genes, respectively (Table 2). The transcriptome assembly containing mouse annotation with percent orthology/homology for each gene is available as [Additional file 1]. Annotation using human reference sequence produced a total of 298,927 (57.19% of total assembled) annotated transcripts in 19,912 transcript families. We found 18,402 seal orthologs and 280,525 seal homologs of human genes (Table 2). The most complete annotation was produced using dog reference sequence, with 356,263 (68.16% of total assembled) total annotated sequences in 24,570 transcript families (Table 2). We found 18,251 seal orthologs and 338,012 homologs of dog genes; 1,630 orthologs and 10,235 homologs were unique to dog and not found in the mouse or human annotations (Figure 1). However, many hits to dog sequences were in poorly annotated genome regions.

**Table 2:** ***Mirounga angustirostris*** **transcriptome annotation statistics**

**Annotated:**	**M. musculus**	**H. sapiens**	**C. familiaris**	**Total**
Orthologs	20,545	18,402	18,251	25,755
Homologs	327,149	280,525	338,012	345,289
Gene families	20,171	19,912	24,570	25,674
Total	347,694	298,927	356,263	359,102
Percent	66.52	57.19	68.16	68.70

The majority of annotated genes were common in mouse, dog, and human datasets (shared orthologs = 12,617, shared homologs = 269,673, Figure 1). We found only 2,763 and 2,233 orthologs that were unique to mouse and human, respectively; 1,670 homologs were mouse-specific and 2,660 were human-specific (Figure 1). We combined the three annotations to produce a total of 359,102 (68.70%) annotated sequences in 25,674 transcript families, with 25,755 *M. angustirostris* orthologs and 345,289 homologs of mouse, dog, and human genes (Table 2, [Additional file 2]).

### Transcriptome filtering

Our assembly produced a vast number of transcripts (half a million), a common issue with *de novo* assemblers. Trinity is especially known for high sensitivity to isoforms [47]. We found that 2,541 (15.56% of total) transcript families contained more than 20 members, with the largest family containing 2,724 isoforms. Isoform overabundance may be due to transcript fragmentation or chimeras created by assembly, and/or complex alternative splicing and high polymorphism common in large vertebrates. Therefore, some isoforms may be assembly artifacts that are not biologically significant [52].

To enable downstream phylogenomic and other analyses, we collapsed gene families that contained more than 20 isoforms by CD-HIT [53,54] clustering with > 95% similarity cutoff followed by removal of redundant transcripts. Only the representative transcript in each cluster was retained. This approach removed 59.95% of redundant isoforms in large gene families, reducing the total number of assembled transcripts from 522,699 to 336,657. The minimum and maximum number of isoforms remaining in filtered families were 3 and 800, respectively. We did not apply a more stringent similarity cutoff as it could result in loss of true splice isoforms. The filtered assembly is available at http://athyra.ged.msu.edu/~preeyano/seal/Mirounga_filtered.fa.gz. We provide both the raw and filtered assembly as filtering may remove true splice isoforms [52]. Therefore, the raw assembly may provide vital information on rare transcripts and novel splice isoforms that may be lost during filtering.

### Functional annotation

**Figure 2:**
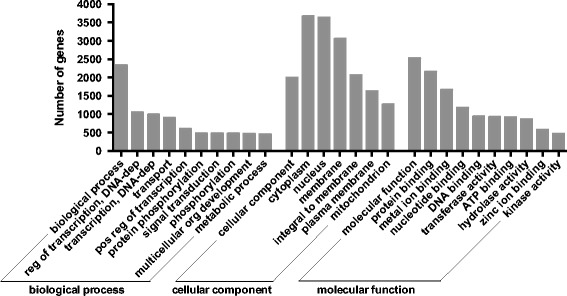
**Top gene ontology (GO) categories represented in the entire transcriptome.** Numbers of genes in the entire *M. angustirostris* transcriptome that mapped to top GO biological process, cellular component, and molecular function terms are shown.

To infer biological function of annotated *M. angustirostris* genes in the muscle transcriptome, we searched for gene ontology (GO, [55]) terms in 14,361 gene families that included orthologs and homologs of mouse proteins. We found that 2,331 genes were associated with biological processes and 2,520 with molecular functions (Figure 2). Of the genes that were associated with a cellular component, 3,660 encoded cytoplasmic proteins, 3,627 encoded nuclear proteins, and 3,048 encoded membrane proteins (Figure 2). Top molecular functions included binding to proteins (n = 2,150 genes), metal ions (n = 1,660), and nucleotides (n = 1,173), and transferase (n = 920), hydrolase (n = 866), and kinase (n = 469) activities (Figure 2). Top biological process categories included transcriptional regulation (n = 1052), transport (n = 899), signal transduction (n = 475), organismal development (n = 469), and metabolic processes (n = 448, Figure 2).

**Figure 3:**
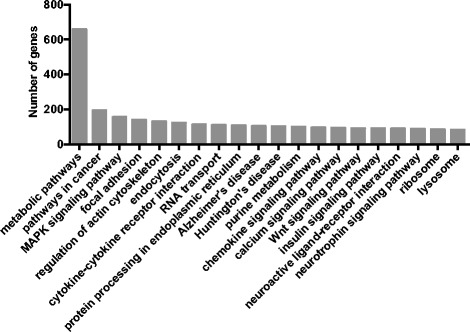
**Top 20 KEGG pathways represented by annotated** ***M. angustirostris*** **homologs and orthologs of mouse genes.**

To identify specific metabolic and signaling pathways encoded in the transcriptome, we mapped mouse-annotated *M. angustirostris* genes to KEGG pathways [56]. The most highly enriched pathways are involved in metabolism (n = 608), cancer (n = 168), MAPK signaling (n = 140), focal adhesion (n = 121), and actin cytoskeleton regulation (n = 112 genes, Figure 3). Enriched pathways also include those involved in immunity (cytokine-cytokine receptor interaction, chemokine signaling pathway, leukocyte transendothelial migration, T-cell receptor signaling pathway, natural killer cell mediated cytotoxicity, B-cell receptor signaling pathway) and response to pathogens (toxoplasmosis, Chagas disease, amoebiasis, bacterial invasion of epithelial cells, leishmaniasis, malaria, staphylococcus aureus infection, and African trypanosomiasis, [Additional file 3]).

*M. angustirostris* transcripts were mapped to specific metabolic pathways that include insulin signaling pathway and Type II diabetes mellitus, citrate cycle, oxidative phosphorylation, unsaturated fatty acid biosynthesis, adipocytokine signaling, and glycolysis/gluconeogenesis (Figure 3, [Additional file 3]). Gene sequences that may be of interest to further elephant seal molecular physiology studies include insulin receptor substrate, resistin, hormone-sensitive lipase, thyroid hormone receptor, nitric oxide synthase, heme oxygenase, hypoxia-inducible factor, xanthine oxidase, and superoxide dismutase, among thousands of others.

### Preliminary differential expression analysis

**Figure 4:**
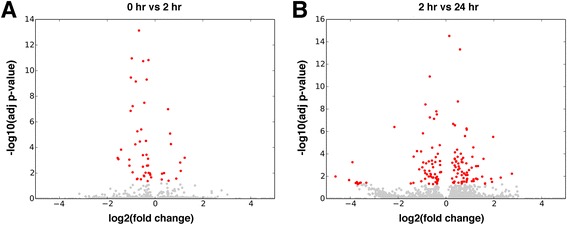
***M. angustirostris*** **genes differentially expressed during an acute stress challenge.** Genes differentially expressed during **(A)** acute stress (baseline (0 hr) versus 2 hr after ACTH administration) and **(B)** stress recovery (2 hr versus 24 hr after ACTH administration) conditions. Log2 fold change for each gene is shown on the x-axis, with -log10 adjusted p-value (false discovery rate < 0.05) on the y-axis. Genes that are differentially expressed at p < 0.05 are shown in red, with all other genes in grey.

**Figure 5:**
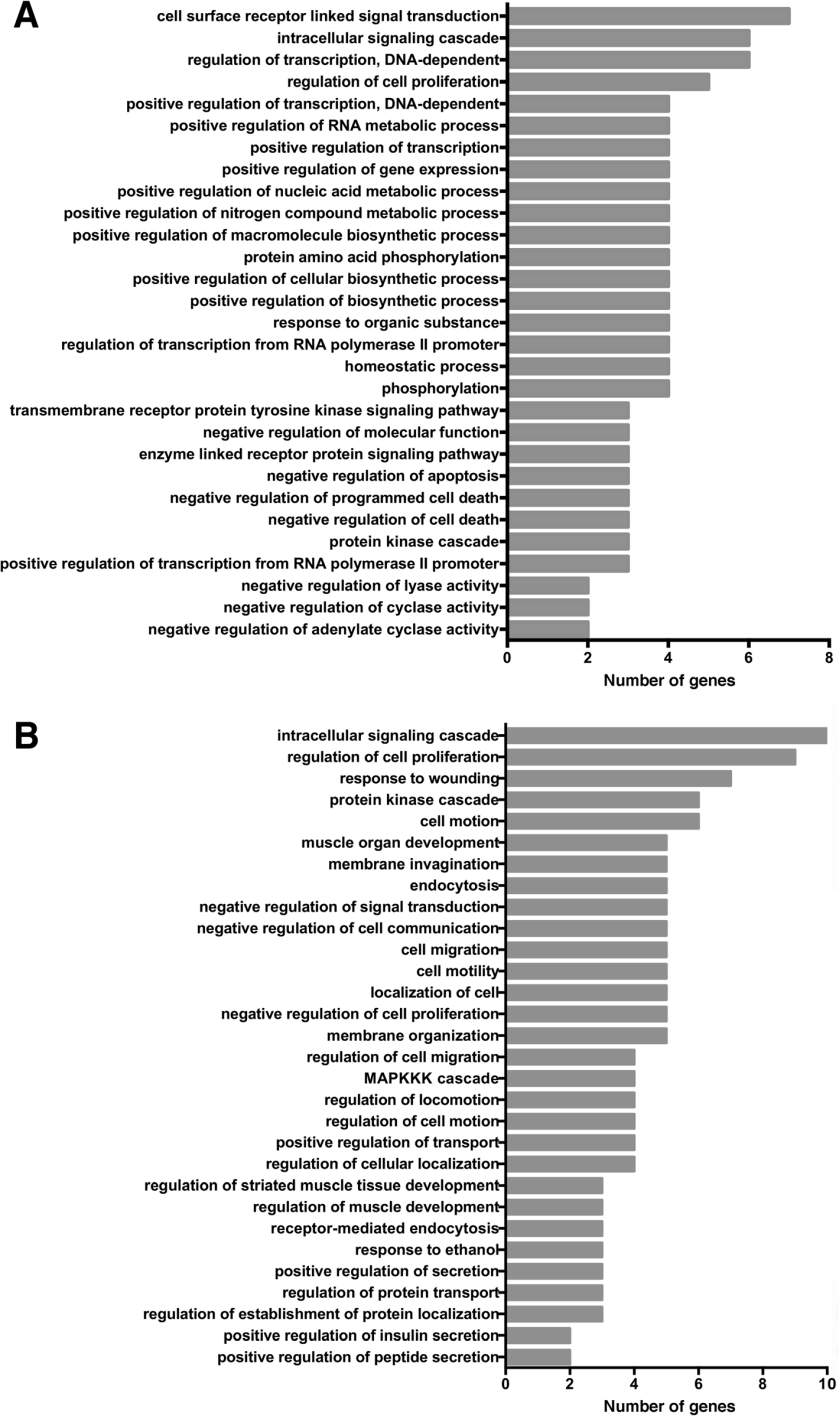
**Gene ontology categories overrepresented in differentially expressed gene datasets.** Top GO biological process terms enriched in gene sets differentially expressed during acute stress **(A)** and recovery **(B)** conditions.

To identify molecular pathways altered in response to an acute stress challenge in *M. angustirostris*, we compared gene expression profiles in libraries from muscle tissue of the three animals before and after ACTH injection. Quality-trimmed reads were mapped to the transcriptome assembly using bowtie. Digital gene expression analysis was conducted using RSEM [57] followed by EBSeq [58]. We found that 52 (0.035%) transcripts were differentially expressed between 0 hr and 2 hr conditions (“acute stress”) at adjusted p-value < 0.05 and false discovery rate (FDR) of 0.05 (Figure 4A). Of these, only 22 transcripts were upregulated or downregulated by at least twofold. Comparison of 2 hr and 24 hr conditions (“stress recovery”) identified 150 (0.100%) differentially expressed genes (adjusted p-value < 0.05, FDR = 0.05), of which 78 were up- or downregulated by at least twofold (Figure 4B). We searched for GO biological process categories that were enriched in the differential expression datasets at p < 0.1. Genes altered within 2 hours of ACTH administration are predominantly involved in transcriptional regulation, cell proliferation, and metabolic signaling (Figure 5A). Transcripts altered during stress recovery mapped to categories such as insulin signaling, muscle tissue development and homeostasis (Figure 5B). This preliminary expression analysis suggests that elephant seal muscle tissue is robust to perturbation and may respond to acute stressors by transiently altering metabolic and tissue remodeling processes.

### Cellular responses to acute stress

In response to HPA axis activation, circulating glucocorticoids bind to receptors in target tissues, which subsequently translocate to the nucleus and serve as transcription factors to influence gene expression. A number of direct glucocorticoid target genes have been identified in skeletal muscle, which include factors that oppose insulin signaling and promote protein catabolism to meet increased energy demands [59]. However, glucocorticoid-induced gene expression has not been extensively studied during acute stress in an *in vivo*, non-pathological context. We found that the most highly upregulated transcript in elephant seal muscle tissue two hours after ACTH administration was CCAAT/enhancer binding protein-δ (*Cebpd*, 4.61-fold upregulation, p = 0.0008), a transcription factor transiently induced by glucocorticoids in other systems. *Cebpd* plays a role in a number of cell processes such as inflammation, cell death and survival, and proteolysis via upregulation of atrogenes [60,61]. Therefore, *Cebpd* is likely an important early regulator of cellular responses to HPA axis activation in elephant seal muscle, and a potential molecular marker of acute stress in this and other marine mammal systems. Interestingly, despite elevation in baseline cortisol over extended fasting periods, elephant seals efficiently spare protein, suggesting that negative feedback mechanisms may operate to suppress glucocorticoids’ proteolytic effects [62]. The transcriptome resource presented here is likely to yield insights on such regulatory mechanisms and will provide a unique molecular resolution of a coordinated stress response *in vivo* in a free-ranging marine mammal study system.

## Conclusions

We generated the first reference sequence for *Mirounga angustirostris* by RNA sequencing of muscle tissue and cloud-based *de novo* transcriptome assembly. We annotated 359,102 *M. angustirostris* transcripts (68.70% of the transcriptome) and have identified thousands of genes involved in key physiological processes such as metabolism, immune response to pathogens, muscle tissue maintenance, and stress. Unannotated transcripts may represent putative novel *M. angustirostris*-specific genes and splice isoforms. This resource provides elephant seal-specific gene sequences, complementing existing metabolite and protein expression studies and enabling future work on molecular pathways regulating adaptations such as fasting, hypoxia, and stress tolerance in muscle tissue. Transcriptional response of muscle to acute stress is limited and may involve alterations in metabolic and immune signaling and muscle tissue maintenance via transcriptional regulators such as *Cebpd*.

## Methods

### Ethics statement

All animal handling procedures were approved by the Sonoma State University Institutional Animal Care and Use Committee and conducted under National Marine Fisheries Service marine mammal permit # 14636. Human protein sequence data used in this study was publicly available at NCBI GenBank and did not require ethics approval.

### Study site and subjects

Juvenile northern elephant seals (*Mirounga angustirostris*) were sampled at Año Nuevo State Reserve (San Mateo County, CA) during their brief annual haulout. This life history stage does not involve breeding or molting, and is therefore the most ‘baseline’ physiological state of the elephant seal accessible to researchers [63]. Animals that had recently arrived at the rookery and were of the same sex (female), age (10 months), and similar body mass (131.7 ± 4.2 kg) and condition were selected to minimize variability.

### Stress challenge experiment and sampling

Study animals were approached while sleeping at the rookery and were immobilized as previously described [63]. Specifically, animals were initially sedated with 1 mg/kg intramuscular injection of tiletamine-zolazepam (Telazol), and sedation was maintained with periodic intravenous doses of ketamine and diazepam (Fort Dodge Laboratories, Fort Dodge, IA). This sedation procedure is known to have no effect on the baseline stress state of elephant seals [24]. Baseline blood samples were obtained via an 18G 3.25- inch needle from the extradural vein within 23.7 ± 8.5 minutes of initial Telazol injection. After subcutaneous injection with 1 ml lidocaine, samples of the left external abdominal oblique muscle were collected using a 6.0 mm diameter biopsy punch (Miltex, York, PA) and immediately frozen in liquid nitrogen. Following initial sample collection (“0 hr”), animals received an intramuscular injection of 0.21 ± 0.01 U/kg corticotrophin LA gel (Westwood Pharmacy, Richmond, VA) on the left side, approximately 1 inch anterior to the initial biopsy site. A second set of blood and tissue samples (on the right, contralateral side of the animal) were collected after 2 hours (“2 hr”). Animals were weighed (MSI tension dynamometer, Seattle, WA), individually marked with rear flipper tags (Dalton Jumbo Roto-tags, Oxon, England) and black hair dye (Lady Clairol, Stamford, CT), and released to resume normal activity [63]. Study subjects were resighted and immobilized 22.7 ± 2.4 hours after initial ACTH injection. A third set of blood samples and right-side tissue samples (“24 hr”) was collected within 16.0 ± 4.0 minutes of Telazol injection as described above.

### RNA isolation

Tissue samples were stored at −80 °C until extraction. In the laboratory, 75–165 mg of muscle tissue were minced with a scalpel on ice, transferred to a glass tissue grinder (Kimble-Chase Kontes Duall, USA), and homogenized with 1 ml of TRIzol Reagent (Life Technologies, USA). RNA was extracted according to the manufacturer’s protocol and purified with the RNeasy mini kit including a 30-minute on-column DNase I digest (Qiagen, USA). RNA was treated with TURBO DNase I (Ambion, Life Technologies, USA) for 30 minutes according to manufacturer’s protocol. Phenol:chloroform:isoamyl alcohol (Affymetrix, USA) extraction was performed to remove DNase I. RNA concentration was quantified on a Qubit fluorometer (Life Technologies, USA).

### Illumina sequencing

Illumina library preparation and sequencing were performed at the UC Davis DNA technologies Core Facility (http://dnatech.genomecenter.ucdavis.edu/) following standard protocols. Total RNA integrity and quantity were evaluated using 2100 Bioanalyzer RNA 6000 kit (Agilent, USA) and Qubit RNA kit (Invitrogen, USA), respectively. RNA samples had integrity values (RIN) of 7.6 - 9.0. Libraries for sequencing were prepared according to TruSeq protocol (Illumina, USA). Specifically, mRNA was isolated from total RNA samples using oligo-d(T)25 magnetic beads (Dynabeads: Invitrogen, USA) and used as template for first-strand cDNA synthesis. After double-stranded (ds) cDNA synthesis, overhang fragments were end-repaired by incubation in the presence of T4 DNA polymerase and Klenow polymerase. The polished fragments were phosphorylated by T4 PNK, followed by the addition of a single ‘A’ base to the 3′ end of the blunt-ended phosphorylated fragments. This ‘A’ base prepared the cDNA fragments for ligation to proprietary adapter oligonucleotides (Illumina, USA) that have a ‘T’ base at their 3′ end. Ligation products were subjected to a final PCR amplification step (8–10 cycles) before library quantification and validation. Individual libraries were prepared with barcode and all nine samples (biological triplicates of 0 hr, 2 hr, and 24 hr samples) were pooled for sequencing on one lane. Sequencing was carried out for 100 cycles on the Illumina HiSeq 2500 platform with paired-end 100 bp reads and library insert size of approximately 500 bp. The average number of reads generated per sample was 28.5 ± 7.5 million. Fastq files were generated using the Illumina Casava pipeline v1.8.2.

### Transcriptome assembly

Sequencing data were assembled using the Eel Pond mRNAseq Protocol (https://khmer-protocols.readthedocs.org/). Analysis was conducted in the cloud using an Amazon EC2 x-large machine (m1.xlarge), except for assembly, for which a 2x-large machine (m2.2xlarge) was required. Downloaded reads were trimmed of sequencing adapters and poor quality sequences using Trimmomatic v0.30 [44] with TruSeq3-PE adapter sequences. Reads with quality scores < 30 and quality base pair percent < 50 were filtered using Fastx toolkit v0.0.13.2. Sequence quality was evaluated by FastQC v0.10.1; per-sequence quality score was 38 for each sample after adapter and quality filtering. One round of digital normalization [13] was performed on all samples to filter redundant reads with coverage and k-mer sizes both set to 20. Assembly was conducted with all nine sequenced samples using Trinity v2013-11-10 [12] with default parameters (k-mer size of 25) and maximum memory size set to 30 GB with 4 CPU. Assembly metrics and alignment statistics were obtained using accompanying Trinity bowtie and samtools scripts. Specifically, bowtie was run using default parameters of maximum number of mismatches (N) = 2, seed length (V) of 28, and maximum total of Phred quality scores at all mismatched positions throughout the alignment (E) = 70.

### Transcript clustering and filtering

Gene families containing > 20 isoforms were selected from the assembly for clustering. Transcripts were clustered using cd-hit-est command in CD-HIT v4.6.1-2012-08-27 [53,54] with > 95% similarity cutoff and only the representative transcripts in each cluster were retained. Scripts used to run clustering are available at: https://github.com/Pinnipeds/Mirounga_transcriptome/tree/scripts.

### Annotation

Putative *M. angustirostris* orthologs were identified by searching for reciprocal best hits (BLASTX and TBLASTN) to mouse (*Mus musculus*, NCBI RefSeq), human (*Homo sapiens,* NCBI RefSeq), and dog (*Canis lupus familiaris*, Ensembl CanFam 3.1.75) peptide reference sequences with E-value cutoff of 10^−3^ as maximum threshold for transcript identity. The reciprocal best hits approach of ortholog detection is known to have low false positive error rate and low sensitivity to E-value cutoff, enabling maximum ortholog recovery at higher E-value thresholds [64]. Best BLASTX hits were calculated with E-value cutoff of 10^−3^ to identify all putative *M. angustirostris* homologs of mouse, human, and dog genes. GO [55] and KEGG [56] analyses were performed with Bioconductor v2.14 goseq package using GO database v2.10.1 and KEGG database v2.10.1. Scripts used for annotation and analysis are available at: https://github.com/Pinnipeds/Mirounga_transcriptome/tree/scripts.

### Gene expression analysis

Transcript mapping and abundance estimation for each sample in the dataset were obtained using bowtie v0.12.7 [49] and RSEM v1.2.8 [57]. EBSeq package v1.5.3 [5] was used for differential expression analysis with false discovery rate set to < 0.05.

## Additional files

Additional file 1:
***M. angustirostris*** **transcriptome assembly with complete annotation (mouse).**
Additional file 2:
***M. angustirostris*** **transcriptome assembly with combined annotation (mouse, human and dog).**
Additional file 3:
**KEGG metabolic pathways overrepresented in** ***M. angustirostris*** **transcriptome.**
